# Towards diagnostic metagenomics of *Campylobacter* in fecal samples

**DOI:** 10.1186/s12866-017-1041-3

**Published:** 2017-06-08

**Authors:** Sandra Christine Andersen, Kristoffer Kiil, Christoffer Bugge Harder, Mathilde Hasseldam Josefsen, Søren Persson, Eva Møller Nielsen, Jeffrey Hoorfar

**Affiliations:** 1 National Food Institute, Technical University of Denmark, Mørkhøj Bygade 19, DK-2860 Søborg, Denmark; 2 Statens Serum Institut, Artillerivej 5, DK-2300 København S, Denmark

**Keywords:** Molecular typing, Culture independent, Next generation sequencing

## Abstract

**Background:**

The development of diagnostic metagenomics is driven by the need for universal, culture-independent methods for detection and characterization of pathogens to substitute the time-consuming, organism-specific, and often culture-based laboratory procedures for epidemiological source-tracing. Some of the challenges in diagnostic metagenomics are, that it requires a great next-generation sequencing depth and unautomated data analysis.

**Results:**

DNA from human fecal samples spiked with 7.75 × 10^1^−7.75 × 10^7^ colony forming unit (CFU)/ml *Campylobacter jejuni* and chicken fecal samples spiked with 1 × 10^2^–1 × 10^6^ CFU/g *Campylobacter jejuni* was sequenced and data analysis was done by the metagenomic tools Kraken and CLARK. More hits were obtained at higher spiking levels, however with no significant linear correlations (human samples *p* = 0.12, chicken samples *p* = 0.10). Therefore, no definite detection limit could be determined, but the lowest spiking levels found positive were 7.75 × 10^4^ CFU/ml in human feces and 10^3^ CFU/g in chicken feces. Eight human clinical fecal samples with estimated *Campylobacter* infection loads from 9.2 × 10^4^–1.0 × 10^9^ CFU/ml were analyzed using the same methods. It was possible to detect *Campylobacter* in all the clinical samples.

**Conclusions:**

Sensitivity in diagnostic metagenomics is improving and has reached a clinically relevant level. There are still challenges to overcome before real-time diagnostic metagenomics can replace quantitative polymerase chain reaction (qPCR) or culture-based surveillance and diagnostics, but it is a promising new technology.

## Background

Culture-independent molecular methods as quantitative polymerase chain reaction (qPCR) and Sanger sequencing are well-established and widely used for detection of foodborne pathogens, typically at the species level. Bacterial isolates are needed for the further characterization and typing below the species level, either by the use of traditional methods or by whole genome sequencing, which is being implemented as the routine procedure in laboratories worldwide [1].

Shotgun metagenomics, i.e. sequencing directly from complex samples, is relatively fast, has the potential to provide genomic information e.g. virulence genes and antibiotic resistance, and the potential to detect all pathogens and mixed infections. In addition, the culture-independency makes it possible to study hard-to-culture and uncultivable organisms. In diagnostic metagenomics as a subset of shotgun metagenomics, detection and typing of pathogens are studied. Despite the potential of diagnostic metagenomics, the method is challenged by sample complexity, sequencing depth and data amount, as well as differences in community composition dependent on sample preparation and deoxyribonucleic acid (DNA) extraction, and the challenge in assembly of whole genomes and connection of e.g. resistance genes to organisms [2].

Diagnostic metagenomics has been investigated in several retrospective studies using different methods for data analysis: *Campylobacter jejuni* was detected in a study of one ill person with diarrhea, using Basic Local Alignment Search Tool (BLAST) [3]. In another metagenomics study, 45 fecal samples from a 2011 outbreak of Shiga-toxigenic *Escherichia coli* in Germany were analyzed, using a de novo approach, an alignment approach, and the tool Metaphlan [4].

In diagnostic metagenomics the sample matrix has a great influence on the accessibility of data on the pathogen. Due to the extensive and complex microbial composition, fecal samples are difficult to analyze. Unbiased sequencing has been successfully used clinically in detection of novel viruses from comparatively low diversity environments in the human body, such as kidney, skin, lungs or cervix [5–7], and detection of bacteria in urine samples [8]. However, finding pathogenic bacteria in the gut, a microbial environment with both a high load of commensals and a high diversity, is certainly much more challenging, and to our knowledge only done retrospectively, e.g. [3, 4].

Here we present a study of the limits and linearity in detection of *Campylobacter* - the most prevalent foodborne bacterial pathogen. First, a robust bioinformatics analysis, including the removal of false positive hits from phage and plasmid DNA, was developed by the use of data sets produced from human and chicken fecal samples spiked with controlled amounts of *Campylobacter jejuni*. Secondly, by the analysis of clinical samples from patients with *Campylobacter* infection, the developed analysis method was used for confirming the obtained theoretical detection limits.

## Methods

### Experimental design

**Fig. 1:**
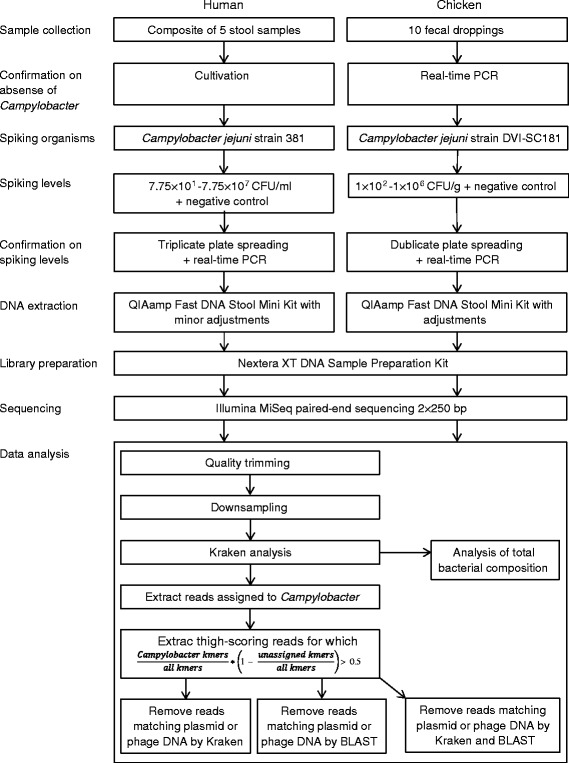
Study design. Flowchart showing workflow in the study and differences between the analysis of human and chicken samples

The laboratory work for this publication was done at two different sites. The human samples were analyzed at Statens Serum Institut, Denmark, and the chicken samples were analyzed at the National Food Institute, Technical University of Denmark. All samples were sequenced at Statens Serum Institut. Hence, there are minor differences between the laboratories in materials and methods used. The experimental setup including the most important differences and the data analysis is seen in Fig. 1.

### Human samples

A total of 22 clinical, *Campylobacter* culture-positive, fecal samples were kindly provided by Bente Olesen at the Department of Clinical Microbiology, Herlev Hospital, Denmark. Five *Campylobacter* culture-negative human fecal samples were pooled and mixed with sterile buffered saline to obtain a pipetable and uniform consistency, and spiked with a liquid culture of *C. jejuni* strain 381 in 10-fold serial dilutions from 7.75 × 10^1^−7.75 × 10^7^ colony forming units (CFU)/ml. A negative control spiked with physiological saline was included. Spiking levels were confirmed by triplicate plate spreading and qPCR. All spiked samples were stored at 4 °C until DNA extraction. DNA from the clinical samples was stored at −18 °C for 3 years before eight samples were sequenced.

### Chicken samples

Ten chicken fecal droppings of approximate 10 g each were collected in a conventional Danish chicken house with no known history of *Campylobacter* infections. The samples were transported at 4 °C to the laboratory, and processed within 2 hours of collection. The fecal droppings were pooled upon confirmation of the absence of *Campylobacter* by qPCR. Subsamples were spiked with a liquid culture of *C. jejuni* strain DVI-SC181 in 10-fold serial dilutions from 1 × 10^2^ to 1 × 10^6^ CFU/g. One subsample was used as a negative control. Spiking levels were confirmed by duplicate plate spreading and qPCR.

### DNA extraction

DNA was extracted from spiked and clinical human fecal samples with QIAamp Fast DNA Stool Mini Kit (QIAGEN, Hilden, Germany) according to the manufacturer’s instructions for pathogen detection, however with the following modifications: In step 14, 110 μl Buffer ATE was used to increase DNA concentration. As starting material, 200 μl of fecal sample was used. DNA was extracted from chicken fecal samples with QIAamp Fast DNA Stool Mini Kit (QIAGEN, Hilden, Germany) according to the manufacturer’s instructions on pathogen detection step 1–4 (inhibition buffer and heat treatment), and on human DNA analysis step 5–14 (larger volumes of reagents) to maximize DNA yield according to [9]. However, in step 14, 100 μl Buffer ATE was used to increase DNA concentration. In addition, based on observation from other studies [10], the starting material was standardized to 0.2 g of fecal sample. DNA was extracted from *C. jejuni* strain 381 and *C. jejuni* strain DVI-SC181 with QIAamp Fast DNA Stool Mini Kit (QIAGEN, Hilden, Germany) according to the manufacturer’s instructions on pathogen detection.

### qPCR

A validated qPCR assay for thermotolerant *C. jejuni*, *C. coli* and *C. lari* based on amplification of a 287 basepair (bp) sequence of the 16S rRNA gene was performed on a Stratagene Mx3005P (ThermoFisher Scientific, Waltham, MA USA) for all samples in the present study [11]. Fluorescence measurements were analyzed with the MxPro-Mx3005P software (version 4.10). The threshold was assigned using the software option background-based threshold; i.e., the standard deviation of all amplifications was determined from cycle five to cycle nine, and this value was multiplied by a background sigma multiplier of ten [12]. Samples were analyzed in duplicate, and every qPCR analysis included a non-template control, a negative control (5 ng of *Escherichia coli* DNA), positive controls (0.5, 0.05 and 0.005 ng of *C. jejuni* DNA) and intern amplification control in all wells to avoid false negative responses. For clinical samples infected with *Campylobacter* the standard curve calculated from spiked human samples was used to estimate the infection load.

### Library preparation and sequencing

Procedures for library preparation and sequencing were identical for all samples. DNA was quantified with Qubit High Sensitivity kit (ThermoFisher Scientific, Waltham, MA, USA), and libraries for sequencing were prepared using the Nextera XT DNA Sample Preparation kit (Illumina, San Diego, CA, USA) according to the manufacturer’s protocol. Paired-end sequencing with 2 × 250 bp was done on Illumina MiSeq (Illumina, San Diego, CA, USA) using V2 Chemistry. The eight spiked human samples were multiplexed and sequenced together on one flow cell, the six spiked chicken samples were multiplexed and sequenced together on one flow cell, and the eight clinical samples were multiplexed and sequenced together on one flow cell.

DNA from *C. jejuni* strain 381 and *C. jejuni* strain DVI-SC181 was paired-end sequenced with 2 × 150 bp multiplexed with 16 bacterial isolates not included in the present study, and sequenced on one flow cell.

### Data analysis

Reads were trimmed and samples downsized using Mothur v.1.31.2 [13]. Reads were trimmed to a minimum length of 50 bp, an average quality of 25 in a sliding window of 50 bases and a minimum quality of the single bases of 10. Reads containing ambiguous bases were removed. Samples were downsized for all samples in a data set to contain the same number of reads as the sample in the set with the fewest reads.

Based on preliminary studies (data not shown) we decided to analyze the data thoroughly using a combination of Kraken [14] and nucleotide BLAST [15]. Kraken is a metagenomic sequence classifier based on exact alignment of k-mers. The Kraken database contains k-mers and corresponding lowest common ancestors. Sequences without matching k-mers in the database are left as unclassified. We analyzed our data using default settings and the standard bacterial database. For comparisons, data were also analyzed by CLARK [16] using the same database as for Kraken and classifying at genus level. Afterwards CLARK hits were analyzed by BLAST and hits to phages and plasmids were removed. For all BLAST analyses we used an e-value cutoff of 10^−60^. Contigs from *C. jejuni* strain 381 and *C. jejuni* strain DVI-SC181 were assembled using CLC Genomic Workbench (QIAGEN Bioinformatics, Aarhus, Denmark), and the genomes were added to the Kraken database. The database was downloaded March 2014.

Based on Kraken reports we extracted all reads assigned to *Campylobacter*. These reads were given a score between 0 and 1 calculated as ${\frac{Campylobacter\  kmers}{all\  kmers}}^{\ast}\left(1-\frac{unassigned\  kmers}{all\  kmers}\right)$. Reads with a score above 0.5 were further analyzed. Hits to plasmids and phages were discarded first by Kraken using the standard bacterial database and then by BLAST using all plasmid and phage sequences available from Genbank in beginning of July 2015 [17]. Only hits to chromosomal DNA were kept and considered as positive hits. For clinical samples, hits with only one BLAST reference were removed before removal of hits to plasmids and phages by BLAST. Simple linear regressions correlating log transformed spiking levels and log transformed final number of Kraken hits were made.

Rarefaction curves of the mean species richness were created based on Kraken reports on down sampled data. The total bacterial composition was described in histograms. Reads identified to phylum level were included in the analysis, whereas unclassified reads and reads only identified above phylum level were left out. All phyla which made up at least 1% of the total bacteria were assigned their own category, the remaining phyla were collected in the category “other assigned”.

## Results

### qPCR, sequencing and data quality

The quality of qPCR results, sequencings and data quality was high enough to support our analyses (Table 1).

**Table 1:** Quality of results

		Human fecal samples	Chicken fecal samples	Clinical fecal samples
qPCR	r^2^	0.993	0.939	0.996
	efficiency	78.3%	79.8%	104%
Whole genome sequencing of spiking organisms	Genomic coverage of assembly	40.2×	113.8×	N/A
	N50	221,548	177,674	N/A
	Number of contigs	281	67	N/A
MiSeq sequencing	Cluster density (clusters/mm^2^)	991	1198	1326
Data quality after trimming	Number of reads (billions)	2.96–5.57	4.27–8.47	2.74–6.20
	Minimum read length	50	50	50
	Maximum read length	251	251	251
	Average read length	217–229	193–232	172–206
Data set size after down sampling	Number of reads (billions)	2.96	4.27	2.74

qPCR efficiency was at the same level for the human and chicken spiking series and clinical samples and all unspiked samples were qPCR negative. MiSeq sequencings yielded cluster densities from 991 to 1326 clusters/mm^2^. Number of reads in datasets after quality trimming varied from 2.74 to 8.47 million reads, with average read lengths from 172 to 232 bases. All dataset were downsized so each sample in a data set contained the same number of reads. The assembly of spiking organisms was of sufficient quality for both organisms.

### Data analysis of human clinical samples

Results from data analysis of clinical samples including infection load determined by qPCR, Kraken hits before and after filtering, and CLARK hits before and after filtering are shown in Table 2.

**Table 2:** Data analysis of clinical samples

Infection load (CFU/ml)	Kraken raw	Kraken final	CLARK raw	CLARK final	Host contamination %
9.2 × 10^4^	757	10	155	81	0.72
5.0 × 10^6^	1940	295	932	159	1.26
2.2 × 10^7^	5129	848	5034	3126	0.10
2.6 × 10^7^	3917	565	3667	2170	0.27
1.0 × 10^8^	3286	362	3122	2048	0.18
2.0 × 10^8^	42,412	20	5625	4146	37.59
4.6 × 10^8^	13,288	2609	13,141	9787	0.12
1.0 × 10^9^	199,512	3439	199,219	14,872	0.15

The clinical samples had estimated infection loads between 9.2 × 10^4^ and 1.0 × 10^9^ CFU/ml. It was possible to detect *Campylobacter* in all samples as they were all positive in both Kraken final and CLARK final. Seven of the samples contained 0.10–1.26% human DNA whereas the last sample contained much more, 37.59%.

### Data analysis of spiked samples

Results from data analysis of spiked samples including Kraken hits before and after filtering and CLARK hits before and after filtering are shown in Table 3.

**Table 3:** Data analysis of spiked samples

Spiking level	Kraken raw	Kraken final	CLARK raw	CLARK final
Human samples				
0	218	0	198	15
7.75 × 10^1^	134	0	128	6
7.75 × 10^2^	168	0	152	8
7.75 × 10^3^	212	0	195	12
7.75 × 10^4^	203	4	187	15
7.75 × 10^5^	172	0	148	8
7.75 × 10^6^	238	50	227	73
7.75 × 10^7^	1097	615	1043	703
Chicken samples				
0	234	2	184	12
1 × 10^2^	256	0	229	12
1 × 10^3^	217	2	183	11
1 × 10^4^	794	423	760	531
1 × 10^5^	569	222	536	290
1 × 10^6^	5976	4141	5923	5018

Higher spiking levels generated more hits to *Campylobacter*, but the number of hits was not proportional to spiking level. The raw Kraken and CLARK analyses showed that there was a “background level” of false positives in all samples including samples not spiked and being qPCR negative. Removal of phage and plasmid DNA reduced the number of false positives markedly, although it was not sufficient to remove all hits in unspiked samples. All spiked human samples contained 0.02% human DNA corresponding to 3541–4064 reads. Chicken samples contained 0.04–0.07% chicken DNA corresponding to 1737–3101 reads.

Detection limits were based on Kraken final hits. For human samples, the unspiked sample was negative as were the samples spiked with 7.75 × 10^1^, 7.75 × 10^2^, 7.75 × 10^3^, and 7.75 × 10^5^ CFU/ml. The positive sample with lowest spiking level was spiked with 7.75 × 10^4^ CFU/ml, which can be seen as a “best case” detection level. A worst case detection limit would be 7.75 × 10^6^ CFU/ml, as all samples with higher spiking levels were positive. For chicken samples, the positive sample with the lowest spiking level was spiked with 1 × 10^3^ CFU/g and had two hits. As all samples with higher spiking levels were positive 1 × 10^3^ CFU/g can be seen as a best case detection limit. However, the negative sample also had two hits, which makes 1 × 10^4^ CFU/g the worst case detection limit.

Linearity was investigated using simple linear regressions correlating log transformed spiking levels and log transformed final number of Kraken hits. For human samples with hits above the best case detection limit (*n* = 3), there were no linear correlation between the number of hits and the corresponding spiking levels (*p* = 0.12). For chicken samples above the best case detection limit (*n* = 4), there were no linear correlation between the number of hits and the corresponding spiking levels (*p* = 0.10).

**Fig. 2:**
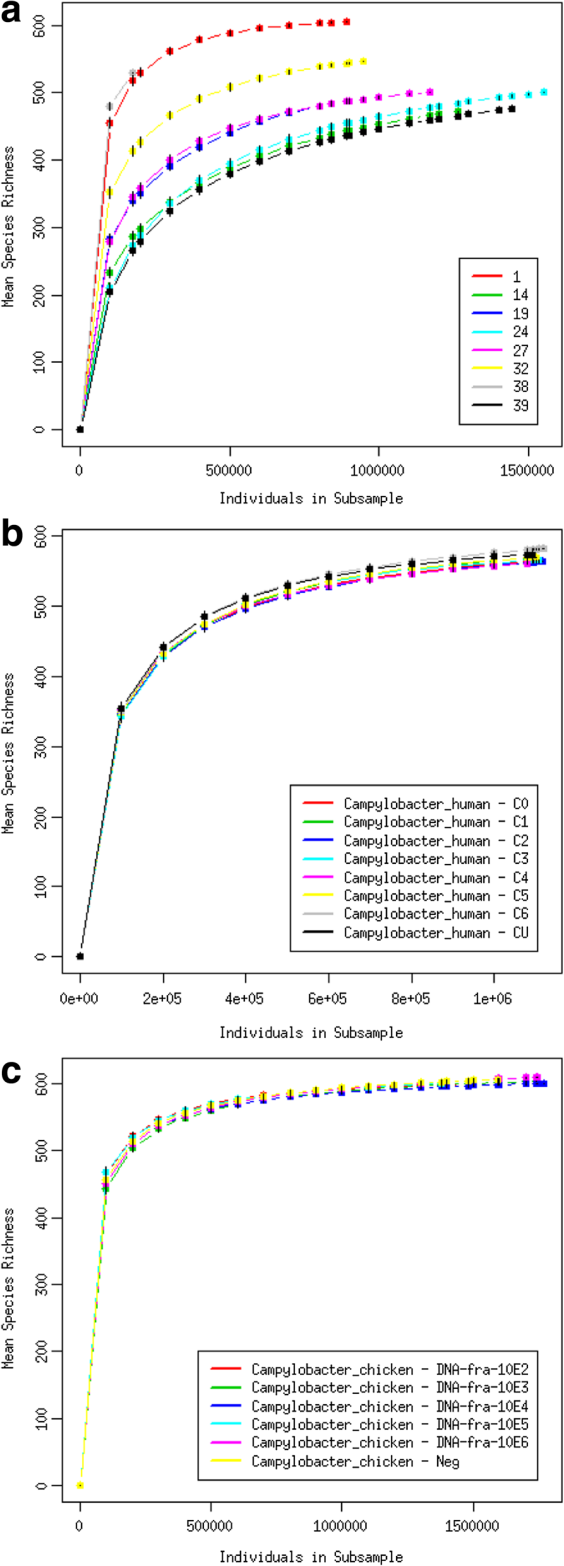
Rarefaction curves of the mean species richness for each subsample. Horizontal axes hold individuals in subsample and vertical axes hold mean species richness. **a** human clinical fecal samples, **b** human spiked fecal samples, **c** chicken spiked fecal samples

Figure 2 shows rarefaction curves for human clinical fecal samples (a), spiked human fecal samples (b) and spiked chicken fecal samples (c). On curves for spiked samples all the subsamples are very close to each other. The curve for chicken samples flatten more of than that for human samples, showing that the chicken subsamples represent the total community a bit better than the human subsamples. The curves for clinical samples are more different. The curves flatten most for the samples 1 and 38 and least for the samples 14, 24 and 39.

**Fig. 3:**
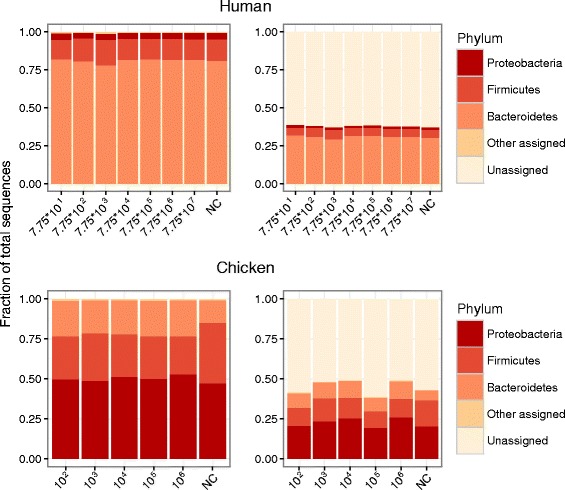
Total bacterial composition. Human samples above, chicken samples below. Left barcharts show the composition of all reads assigned to phylum level. Right barcharts show the composition including unassigned reads. NC = negative control

Analyses of the total bacterial composition (Fig. 3) were based on the Kraken reports. For human feces approximately 40% of the reads were classified and the main phyla were *Bacteroidetes* and *Firmicutes*. For chicken feces approximately half of the reads were classified and the main phyla were *Proteobacteria*, *Firmicutes* and *Bacteroidetes*.

## Discussion

There are three main findings in the present study: First, it was possible to detect *Campylobacter* in eight human clinical fecal samples with estimated infection loads of 9.2 × 10^4^–1.0 × 10^9^ CFU/ml. Second, based on spiked human fecal samples, a best case detection limit was set to 7.75 × 10^4^ CFU/ml and a worst case detection limit was set to 7.75 × 10^6^ CFU/ml. Based on spiked chicken fecal samples, a best case detection limit was set to 1 × 10^3^ CFU/g and a worst case detection limit was set to 1 × 10^4^ CFU/g. Finally, false positive hits in unspiked, qPCR-negative samples were markedly reduced or completely removed by removal of phage and plasmid DNA.

The infection load in the clinical samples varied with a factor of 10^4^. This is a great span and still we were able to convincingly detect *Campylobacter* in all samples. The optimistic detection limit is just below the lowest clinical infection load, making it reliable that this is actually the true detection limit, although the sample spiked with 7.75 × 10^5^ CFU/ml has no Kraken final hits. We have no explanation why this sample has not final Kraken hits, as it is qPCR positive with a Ct-value that fits well with the other samples in the spiking series.

The detection limits for human and chicken fecal samples were quite different. This may be because the human intestinal bacterial community is more diverse or because human feces contain a higher number of bacteria per gram of feces. A third possibility is, that we see this difference due to down sampling, as the human samples were down sampled to 2.96 billion reads and the chicken samples to 4.27 billion reads. The rarefaction curves in Fig. 2 show that the down sampled chicken samples describe the bacterial diversity a bit better than the down sampled human samples. However, this sequencing depth was enough to detect *Campylobacter* in all clinical samples which were down sampled to 2.74 billion reads. To gain the same sensitivity as qPCR approximately ten times more data from each sample is needed for both matrices.

Despite the same fecal matrix was used for spiking throughout a spiking series, the fraction of reads possible to assign and the bacterial composition on phylum level varied more than expected among the spiking levels. However, all the samples in a spiking series were very similar in the rarefaction curves. Probably the observed variation in background is due to the nature of next-generation sequencing techniques.

In the present study it was not possible to find a linear correlation between spiking level and reads originating from the spiking organism. If higher spiking levels had been included in the study it might have been possible to find a linear correlation, as the hits in the two highest spiking levels in each spiking series differed with a factor 10, as the spiking did. It is unknown why we do not see this linearity, but it may be due to the non-linear nature of metagenomic data [18], which should rather not be down sampled and instead be modelled as a mixture distribution. With the consistent background of the spiked samples, the non-linearity of detected spiked reads is troubling.

Host contamination was not considered a problem for the spiked samples, as it comprised a very small part of the DNA. This was also true for most of the clinical samples, except the one with a host contamination of 38%. For this sample the host contamination may have influenced the result of the analysis of bacterial content, as the host DNA was not removed from the data neither before nor after the down sampling. In future studies of clinical samples it may be recommendable to remove host DNA before analyzing the bacterial DNA.

When interpreting our results from the data analysis it became clear, that some hits were false positives, because *Campylobacter* was detected in high numbers in samples that were not spiked qPCR negative. We were able to remove these false positive hits in the Kraken analysis, and to reduce them in the CLARK analysis. In general the raw Kraken and CLARK results were in agreement with each other for all three dataset. The raw Kraken analysis was improved by scoring hits and removal of phages and plasmids by Kraken and BLAST. This removal of false positive hits substantially improved the confidence in the Kraken final results. The CLARK analysis was only improved by removal of phages and plasmids by BLAST, and this was not enough to remove all false positive hits. This makes it clear, that it is important to be familiar with the tools chosen for data analysis, and that it may be necessary to adjust or add to these tools.

## Conclusions

The sensitivity of diagnostic metagenomics is improving and has reached a clinically relevant level. For diagnostic metagenomics to become useful in surveillance and diagnostics of food borne diseases it is important to continuously improve reliable and robust bioinformatic tools for data analysis. While we at present do not recommend replacing current qPCR or culture-based surveillance and diagnostic programs, the present study is a step in that direction.

## Abbreviations

BLAST: Basic local alignment search tool
bp: Basepair
CFU: Colony forming unit
DNA: Deoxyribonucleic acid
qPCR: Quantitative polymerase chain reaction
